# Ameliorative effect of chitosan nanoparticles against carbon tetrachloride-induced nephrotoxicity in Wistar rats

**DOI:** 10.1080/13880209.2022.2136208

**Published:** 2022-10-28

**Authors:** Yousra A. Nomier, Saeed Alshahrani, Mahmoud Elsabahy, Gihan F. Asaad, Azza Hassan, Walaa A. El-Dakroury

**Affiliations:** a Pharmacology and Toxicology Department, Pharmacy College, Jazan University, Jazan, Saudi Arabia; b School of Biotechnology and Science Academy, Badr University in Cairo, Badr City, Cairo, Egypt; c Department of Chemistry, Texas A&M University, College Station, TX, USA; d Department of Pharmacology, Medical Research and Clinical Studies Institute, National Research Centre, Giza, Egypt; e Pathology Department, Faculty of Veterinary Medicine, Cairo University, Giza, Egypt; f Department of Pharmaceutics and Industrial Pharmacy, Faculty of Pharmacy, Badr University in Cairo, Badr City, Cairo, Egypt

**Keywords:** Renoprotective, CCl4, drug delivery

## Abstract

**Context:**

Chitosan is a biocompatible polysaccharide that has been widely exploited in biomedical and drug delivery applications.

**Objective:**

This study explores the renoprotective effect of chitosan nanoparticles *in vivo* in rats.

**Materials and methods:**

Chitosan nanoparticles were prepared via ionotropic gelation method, and several *in vitro* characterizations were performed, including measurements of particle size, zeta potential, polydispersity index, Fourier transform-infrared spectroscopy, differential scanning calorimetry, and transmission electron microscopy (TEM) imaging. Wistar rats were divided randomly into four groups; negative control, CCl_4_-induced nephrotoxicity (untreated), and two groups receiving CCl_4_ + chitosan NPs (10 and 20 mg/kg) orally for 2 weeks. The renoprotective effect was assessed by measuring oxidative, apoptotic, and inflammatory biomarkers, and via histopathological and immunohistochemical examinations for the visualization of NF-κB and COX-2 in renal tissues.

**Results:**

Monodisperse spherical nanosized (56 nm) particles were successfully prepared as evidenced by dynamic light scattering and TEM. Oral administration of chitosan nanoparticles (10 and 20 mg/kg) concurrently with CCl_4_ for 2 weeks resulted in 13.6% and 21.5% reduction in serum creatinine and increase in the level of depleted reduced glutathione (23.1% and 31.8%), respectively, when compared with the positive control group. Chitosan nanoparticles (20 mg/kg) revealed a significant (*p* ˂ 0.05) decrease in malondialdehyde levels (30.6%), tumour necrosis factor-α (33.6%), interleukin-1β (31.1%), and caspase-3 (36.6%).

**Conclusions:**

Chitosan nanoparticles afforded significant protection and amelioration against CCl_4_-induced nephrotoxicity. Thus, chitosan nanoparticles could afford a potential nanotherapeutic system for the management of nephrotoxicity which allows for broadening their role in biomedical delivery applications.

## Introduction

Chitosan, a biodegradable polysaccharide, is obtained through the partial deacetylation of chitin, a structural component in the shells of shrimps and crustaceans (Kyzas and Bikiaris 2015). Chitosan is widely used in the food industry and in different biomedical applications due to its physical and chemical characteristics (e.g., biocompatibility, safety, cost-effectiveness, mucoadhesive, gelling properties, etc.). Several studies exploited chitosan in preparation of drug delivery systems (e.g., nanocomposites, nanofibres, beads, hydrogels, etc.) (El-Husseiny et al. 2022), thus benefiting chitosan’s inherent antibacterial, anti-inflammatory, antioxidant, and haemostatic properties (Ali and Ahmed 2018). For example, a study by our group demonstrated improved haemostasis and enhanced survival rates using chitosan/kaolin nanoparticles (NPs), compared with conventional dressings and QuikClot^®^ Combat Gauze (Elsabahy and Hamad 2021). A study by Abdel-Wahhab et al. (2017) revealed that administration of chitosan NPs resulted in a significant increase in antioxidant enzymes (i.e., glutathione peroxidase and superoxide dismutase) and reduced oxidative stress marker, malondialdehyde, in rats that received ochratoxin A-contaminated diet. Moreover, chitosan NPs were found to possess anticancer activity (Feng et al. 2013; Subhapradha and Shanmugam 2017) and to augment the activity of anticancer drugs (Anitha et al. 2014; Kandra and Kalangi 2015; Almutairi et al. 2020). Free methylglyoxal or methylglyoxal conjugated chitosan nanoparticles showed antifungal activity against an isolate of *Candida albicans* that did not respond to fluconazole therapy. Chitosan also shows an antifungal activity against *C. albicans* (Khan et al. 2020). Due to the well-reported antioxidant and anti-inflammatory effects of chitosan, we explored the ability of chitosan NPs to protect against CCl_4_-induced nephrotoxicity.

Carbon tetrachloride (CCl_4_) is an organic compound widely used in the manufacturing of cleaning agents and solvents, and in the synthesis of chlorofluorocarbons (Unsal et al. 2021). However, CCl_4_-induced nephrotoxicity may be due to oxidative stress and free radicals which is mediated by cytochrome P450 located in the renal proximal tubules (Ronis et al. 1998). Proinflammatory cytokines possess a pivotal role in inflammation and immunity as well as cellular proliferation, migration, and adhesion. Interleukins and tumour necrosis factors are involved in the upregulation of inflammatory responses (Hamid et al. 2017). CCl_4_ was also implicated in cell apoptosis which leads to various alterations in cellular morphology such as cell shrinkage, DNA fragmentation, and mRNA decay (Safhi 2018). Cellular apoptosis is initiated by the activation of caspase 9 (initiator caspase) followed by the activation of caspase 3 (executioner caspase) causing cellular death due to protein degradation (Rudel 1999).

Previous studies reported noticeable anti-inflammatory and antioxidant effects of chitosan in a glycerol-induced nephropathy (Xing et al. 2014) and gentamicin-induced nephrotoxicity (Chou et al. 2015) animal models. Chitosan nanoparticles are well-known for biocompatibility and high stability of chitosan nanoparticles (Nagpal et al. 2010). However, it has been reported that after exposure to chitosan nanoparticles, zebrafish embryos displayed signs of apoptosis, probably due to oxidative stress induced by increased intracellular formation of reactive oxygen species (Hu et al. 2011). Surface area, surface charge, coating and exposure period are considered as significant factors in dictating nanoparticle toxicity. In the current study, sodium alginate chitosan NPs will be prepared and characterized using dynamic light scattering, Fourier transform-infrared spectroscopy (FTIR), differential scanning calorimetry (DSC), and transmission electron microscopy (TEM). Then, the potential mechanism of the ameliorative effect of chitosan NPs will be assessed in rat model of CCl_4_-induced nephrotoxicity.

## Materials and methods

Chitosan medium molecular weight (190–310 kDa, deacetylation degree 75–85%) was purchased from Sigma Aldrich (St. Louis, MO, USA). Sodium alginate was obtained from Himedia Co. (Mumbai, India). Carbon tetrachloride, thiobarbituric acid, and 5,5-dithiobis-2-nitrobenzoic acid were purchased from Sigma-Aldrich. Absolute alcohol, glacial acetic acid, and all other chemicals were of analytical grades and were used without further modification.

### Preparation of chitosan nanoparticles

Nanoparticles comprising sodium alginate and chitosan (6 mg/mL) were prepared according to the previously established method using ionotropic gelation (El-Shafei et al. 2021). Briefly, chitosan was dissolved in 2% acetic acid at a concentration of 12 mg/mL, and the pH of the solution was adjusted to 4.8 using 1 N NaOH. The alginate solution (6 mg/mL) was prepared in distilled water, the pH was adjusted to 5.2, and was kept at room temperature overnight. Then, chitosan solution was added dropwise into the alginate solution under stirring at 1000 rpm. Afterward, the formulation was sonicated for 15 min using a probe sonicator (Branson, Sonifier 250, Mexico) to reduce particle size.

### Dynamic light scattering studies

Mean particle size (average diameter), zeta potential, and size distribution (polydispersity index, PDI) of diluted freshly prepared chitosan NPs were measured using a Zetasizer Nano ZS (Malvern Instruments, Worcestershire, UK) at a scatter angle of 90° and a temperature of 25 °C. The chitosan NPs were diluted to a 100-fold with double distilled water prior to measurements (Zewail et al. 2022).

### Fourier transform-infrared spectroscopy

FTIR spectra of chitosan, sodium alginate, and freshly prepared chitosan NPs were recorded using FTIR spectrophotometer (IR-8400s, Shimadzu, Japan). Samples were blended with potassium bromide (spectroscopic grade), and compressed into discs by hydraulic press before scanning from 4000 to 400 cm^−1^ .

### Differential scanning calorimetry

The thermal properties of chitosan, sodium alginate and freshly prepared chitosan NPs were assessed, using DSC (TA Q200, USA). Samples (2 mg) were placed in aluminium pans and heated at a rate of 10 °C/min, over a temperature range of 25–350 °C, using a dry nitrogen gas at a flow rate of 50 mL/min.

### Transmission electron microscopy

The surface morphology of chitosan NPs was visualised by TEM (JOEL JEM-2100, Tokyo, Japan). A droplet of the nanoparticles was placed onto a carbon-coated copper grid and dried. An acceleration voltage of 200 kV was applied for TEM visualization (magnification power = 30,000). The number-averaged hydrodynamic diameter of chitosan NPs was determined by measuring particles randomly selected from TEM images using Image J (NIH, v. 1.50i).

### Pharmacological studies

#### Animals

Twenty-four female Wistar rats (150–250 g) were obtained from the animal breeding unit at the National Research Centre (Cairo, Egypt). Rats were kept in clean polypropylene cages maintained at 25 ± 2 °C with 12 h light–dark cycles. Food and water were freely accessible to animals. The experiments and treatment of the rats were approved by the ethical committee of Faculty of Pharmacy, Badr University in Cairo, Cairo, Egypt (approval no: PT-108-A) and in harmonization of the Guidelines of the US National Institute of Health policies.

#### Animal grouping

Twenty-four adult female rats were divided randomly into four groups (6 animals per group). Group 1 received no treatment (negative control), whereas Group 2 received CCl_4_ [1.5 mL/kg dissolved in paraffin oil (1:1 w/v)] twice a week for 2 weeks, via intraperitoneal (IP) injection (Hassan et al. 2017). Groups 3 and 4 received chitosan NPs at doses of 10 and 20 mg/kg for 2 weeks and concurrently injected with CCl_4_ as in group 2. Blood samples were taken after 48 h post last injection of CCl_4_ from the retro-orbital plexus under anaesthesia (ketamine, 20 mg/mL; intramuscular) for the measurement of creatinine level. Animals were then sacrificed by cervical dislocation under anaesthesia and both kidneys from all animals were excised. One kidney was kept at −80 °C for determination of inflammatory cytokines [interleukin-1 (IL-1β) and tumour necrosis factor-alpha (TNF-α)], oxidative stress biomarkers [reduced glutathione (GSH) and malondialdehyde (MDA)], and apoptotic biomarker, caspase-3. The other kidney was kept in formalin (10%) for histopathological and immunohistochemical assessments.

#### Preparation of blood samples

Blood samples were collected from retro-orbital plexus under anaesthesia (ketamine, 20 mg/mL, intramuscular). Serum was then separated using a high-speed centrifuge (MPW-120 homogeniser, Med instruments, Warsaw, Poland) at 1500 rpm for 10 min.

#### Preparation of tissue samples

Kidney sections were homogenised using MPW-120 homogeniser (Med Instruments,Warsaw, Poland) to prepare 20% homogenate in phosphate buffer. The homogenates were centrifuged for 5 min at 5000 rpm using a cooling centrifuge (Sigma Laborzentrifugen, GmbH, Osterode am Harz, Germany). The supernatant was kept at −80 °C till analyses of inflammatory cytokines, oxidative, and apoptotic biomarkers. All results are expressed per mg protein.

#### Determination of serum creatinine

Creatinine was determined in serum using a commercially available kit according to the manufacturer’s instructions (Biodiagnostic, Cairo, Egypt).

#### Determination of oxidative stress markers

GSH was determined according to Ellman (1959) and the optical density (OD) was measured spectrophotometrically at a wavelength = 412 nm. MDA was determined according to Ohkawa et al. (1979) and the OD was measured spectrophotometrically at a wavelength = 532 nm.

#### Determination of inflammatory cytokines

IL-1β and TNF-α were determined using IL-1β and TNF-α kit for rats (R&D Systems, Minneapolis, MN, USA) in kidney homogenates. The OD is measured spectrophotometrically at 450 nm using a spectrophotometer (Molecular Devices, San Jose, CA, USA).

#### Determination of the apoptotic marker caspase-3

Caspase-3 was measured in kidney homogenate using a caspase-3 ELISA kit for rats (CUSBIO, Houston, TX, USA). The absorbance was measured at a wavelength of 450 nm using a spectrophotometer (Molecular Devices, San Jose, CA, USA).

### Histopathological examinations

Kidneys of all animals were fixed in 10% neutral formalin and routinely processed as described by Knodell et al. (1981). Tissue sections of 5 µm thickness were stained with H&E for histopathological examinations. The histopathological parameters for renal damage assessment were vacuolar degeneration and swelling of renal tubular epithelium and basophilic pyknotic nuclei. For each group, 10 random lesions were assessed for renal damage at low power field lens.

### Immunohistochemical analysis

Immunohistochemical staining of renal tissues for the visualization of NF-κB and COX-2 was carried out according to the method by Abd Eldaim et al. (2020). Briefly, the formalin-fixed renal sections were deparaffinized, hydrated in alcohol solutions, and incubated in 3% H_2_O_2_. The sections were then incubated with rabbit polyclonal anti-NF-κB (Abcam, Cambridge, UK) and rabbit polyclonal anti-COX-2 (Abcam) as primary antibodies. The immune response was visualized by using diaminobenzidine (Sigma Aldrich). Immunohistochemical expressions of NF-κB and COX-2 in the renal tissues were assessed in ten random high-power fields (40×) according to the percentage of immune positive cells per a high-power field, as reported by Asaad et al. (2021).

### Statistical analysis

Data were presented as mean ± SD. Statistical analysis was carried out by one-way analysis of variance (ANOVA), followed by Tukey’s test for multiple comparisons. *p* < 0.05 was considered significant. For data analysis and graph presentations, GraphPad Prism software version 5 (San Diego, CA, USA) was used.

## Results

### Dynamic light scattering studies

The particle size of chitosan NPs was nanometric (56 ± 18 nm). Positive zeta potential of ca. 32.5 ± 1.4 mV was recorded, indicating that the NPs are physically stable. The PDI was taken as a measure for the dispersity and homogeneity of the NPs (Zewail et al. 2021). The NPs showed a PDI value of 0.41 ± 0.05.

### Transmission electron microscopy

**Figure 1.**
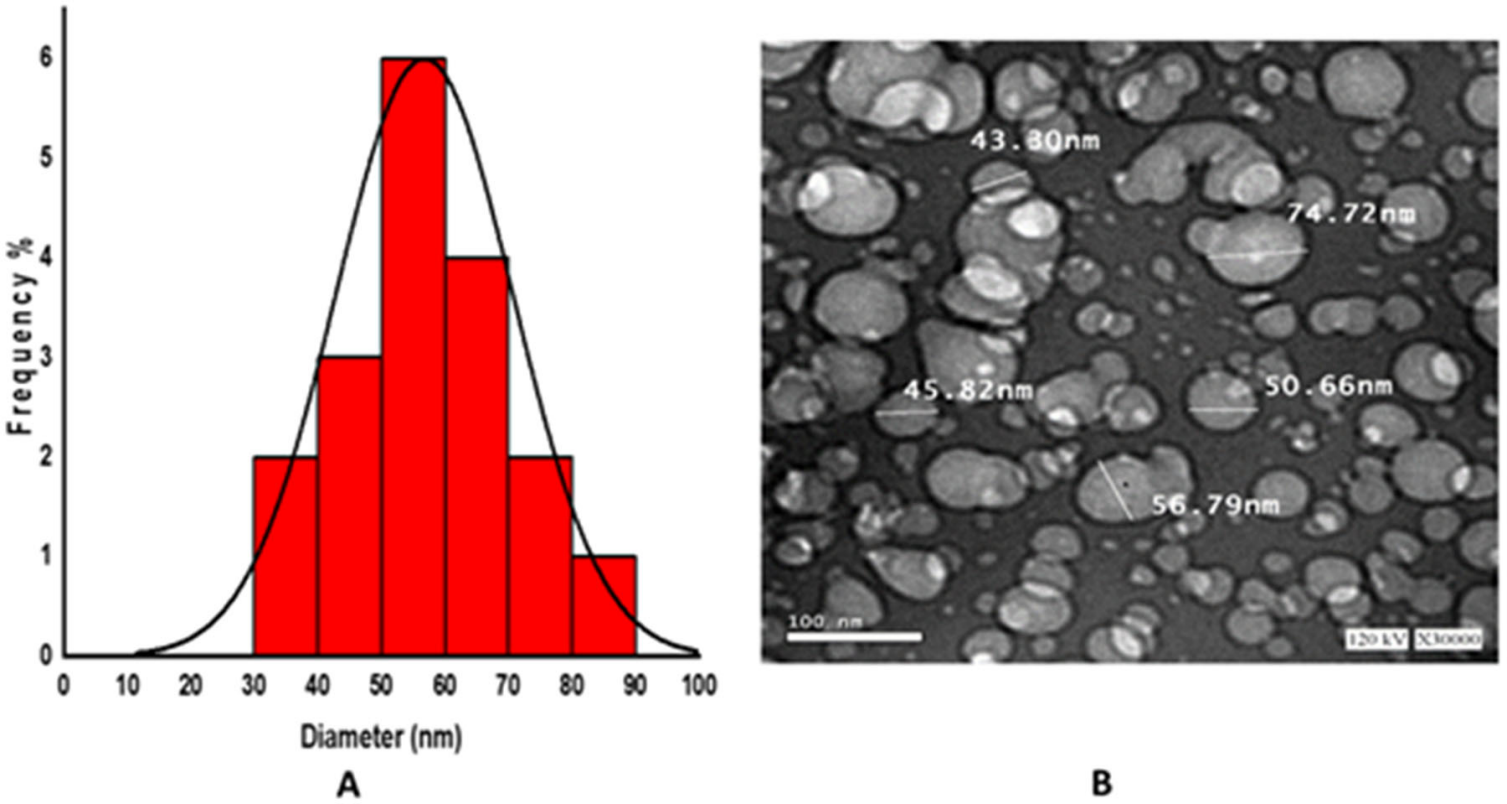
Histogram of the number-averaged hydrodynamic diameter of chitosan NPs (A), and transmission electron microscopy image of the chitosan NPs (B, scale bar = 100 nm).

TEM image and histogram of the size distribution of chitosan NPs are demonstrated in Figure 1A and B. TEM image of the chitosan NPs showed spherical NPs with moderate size distribution (Figure 1(B)). The particle size of the TEM ranged from 43 to 84 nm, in agreement with the particle size obtained from dynamic light scattering. The number-averaged hydrodynamic diameter of chitosan NPs is represented in a histogram and demonstrates a unimodal size distribution (Figure 1(A)).

### Fourier transform-infrared spectroscopy

**Figure 2.**
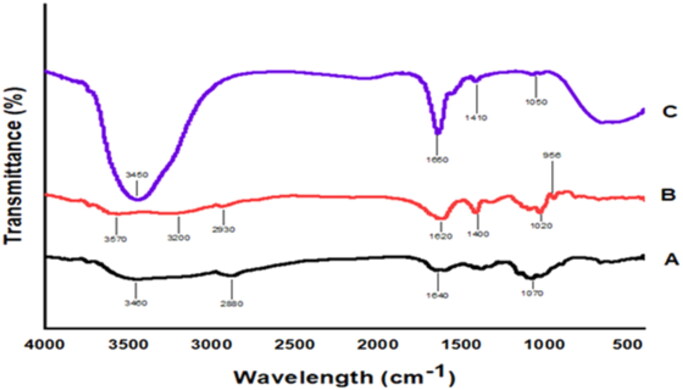
FTIR spectra of (A) chitosan, (B) sodium alginate, and (C) chitosan NPs.

FTIR spectra of chitosan, sodium alginate, and chitosan NPs are shown in Figure 2. The spectrum of chitosan (Figure 2(A)) showed peak at 3448.7 cm^−1^ that corresponds to a hydroxyl group (OH) interfering with (NH group stretching), 2877.4 cm^−1^ for aliphatic CH group, 1650.28 cm^−1^ related to C = O carbonyl group of amides, 1383.52 cm^−1^ for C–O in alcoholic group and 1076.48 cm^−1^ for OH group stretching. The sodium alginate spectrum (Figure 2(B)) revealed characteristic functional peaks at 3241–3561 cm^−1^ allied with OH group expansion, 945 cm^−1^ correlated to uronic acid, 815 cm^−1^ for mannuronic acid, and 2943 cm^−1^ for CH_2_ stretching. The spectrum of chitosan NPs (Figure 2(C)) exhibited visible band-shifts and distinction in intensities of bands. Hydroxyl stretching peaks of chitosan and sodium alginate became wider and the position of the peak was shifted to 3436.65 cm^−1^, while the peak that represents the carbonyl group of amides was maximised, and its intensity increased.

### Differential scanning calorimetry

**Figure 3.**
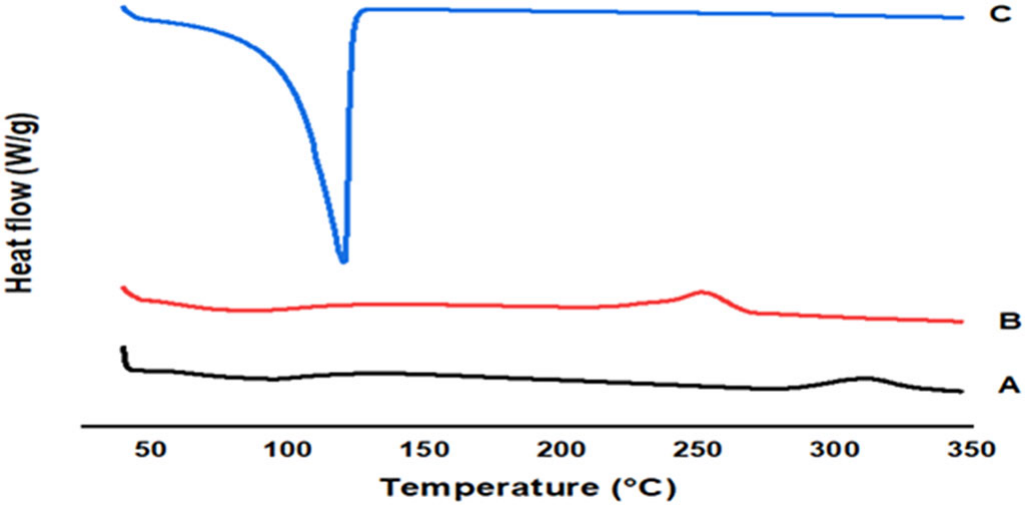
DSC thermogram of (A) chitosan, (B) sodium alginate, and (C) chitosan NPs.

Thermograms of chitosan, sodium alginate and chitosan NPs are demonstrated in Figure 3. DSC thermogram of chitosan (Figure 3(A)) showed an initial endothermic peak at 56.9 °C with heat enthalpy of 126.2 J/g and a higher exothermic peak at 311.9 °C with heat enthalpy of 151.7 J/g. Thermogram of sodium alginate showed an initial endothermic peak at 86.9 °C and a higher exothermic peak at 252.1 °C with heat enthalpy of 222.7 J/g (Figure 3(B)). Thermogram of chitosan NPs (Figure 3(C)) revealed the disappearance of characteristic peaks of chitosan and sodium alginate. The characteristic endothermic peaks for chitosan and alginate were shifted to 100.7 °C with higher intensity and heat of enthalpy of 2193 J/g.

### Determination of serum creatinine

**Figure 4.**
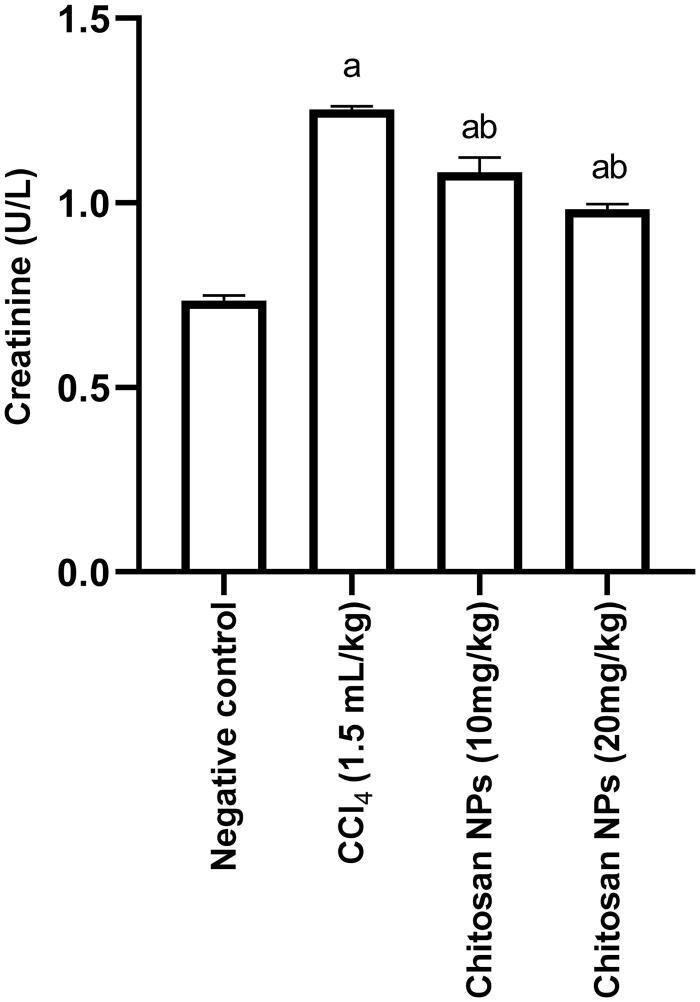
Effect of chitosan NPs (10 and 20 mg/kg) on serum creatinine levels in CCl_4_-induced nephrotoxicity in rats. Data are expressed as mean ± SD. Statistical analyses were performed by one-way ANOVA, followed by Tukey’s *post hoc* test. ^a^*p* < 0.05: statistically significant from the negative control and ^b^*p* < 0.05: statistically significant from the positive CCl_4_ control. CCl_4_: carbon tetrachloride.

IP injection of CCl_4_ [1.5 mL/kg dissolved in paraffin oil (1:1 w/v)] twice daily over 14 days showed a significant (*p* < 0.0001) increase in serum creatinine level (1.25 ± 0.008 U/L) when compared with the control negative group (0.74 ± 0.013 U/L). Groups that received chitosan NPs at doses of 10 and 20 mg/kg, while receiving CCl_4_ over 14 days concurrently, had a significant decrease in serum creatinine [1.08 ± 0.040 U/L (*p* = 0.0002) and 0.98 ± 0.014 U/L (*p* < 0.0001)], respectively, when compared with the positive control group (Figure 4).

### Determination of oxidative stress biomarkers (GSH and MDA)

A strong correlation between GSH and MDA levels (oxidative stress biomarkers) has been previously reported, the higher the MDA level, the lower is the level of GSH (El-Dakroury et al. 2022). IP injection of CCl_4_ resulted in a significant decrease in renal GSH concentration (0.43 ± 0.008 nmol/mg protein) (*p* < 0.0001) when compared with the negative control group (0.61 ± 0.029 nmol/mg protein). The group that received chitosan NPs at doses of 10 and 20 mg/kg, while receiving CCl_4_ over 14 days concurrently, had a significant increase in GSH concentration [0.52 ± 0.016 nmol/mg protein; (*p* = 0.0074) and 0.56 ± 0.016 nmol/mg protein; (*p* = 0.0003)]), when compared with the positive control group.

**Figure 5.**
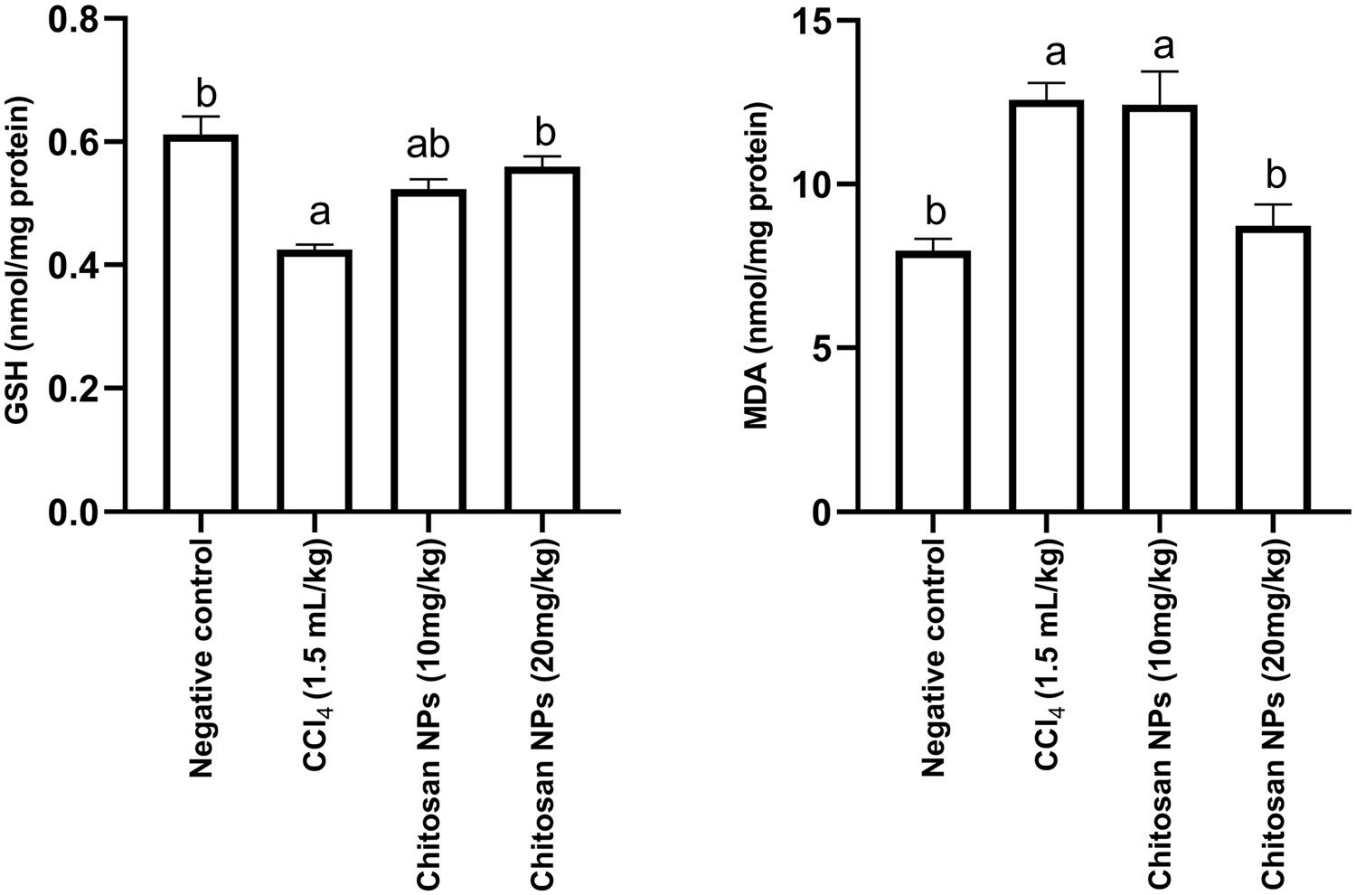
Effect of chitosan NPs (10 and 20 mg/kg) on tissue GSH and MDA concentrations in CCl_4_-induced nephrotoxicity in rats. Data are expressed as mean ± SD. Statistical analyses were performed by one-way ANOVA, followed by Tukey’s *post hoc* test. ^a^*p* < 0.05: statistically significant from the negative control group and ^b^*p* < 0.05: statistically significant from the CCl_4_ control group. CCl_4_: carbon tetrachloride; GSH: reduced glutathione; MDA: malondialdehyde.

IP injection of CCl_4_ resulted in a significant (*p* = 0.0006) increase in renal MDA concentration (12.58 ± 0.52 nmol/mg protein) when compared with the negative control group (7.97 ± 0.37 nmol/mg protein). Only the group that received chitosan NPs (20 mg/kg) had a significant (*p* = 0.0035) decrease in MDA concentration (8.73 ± 0.64 nmol/mg protein), when compared with the positive control group, and no significant (*p* = 0.8533) difference from the negative control group (Figure 5).

### Determination of inflammatory cytokines (IL-1β and TNF-α)

**Figure 6.**
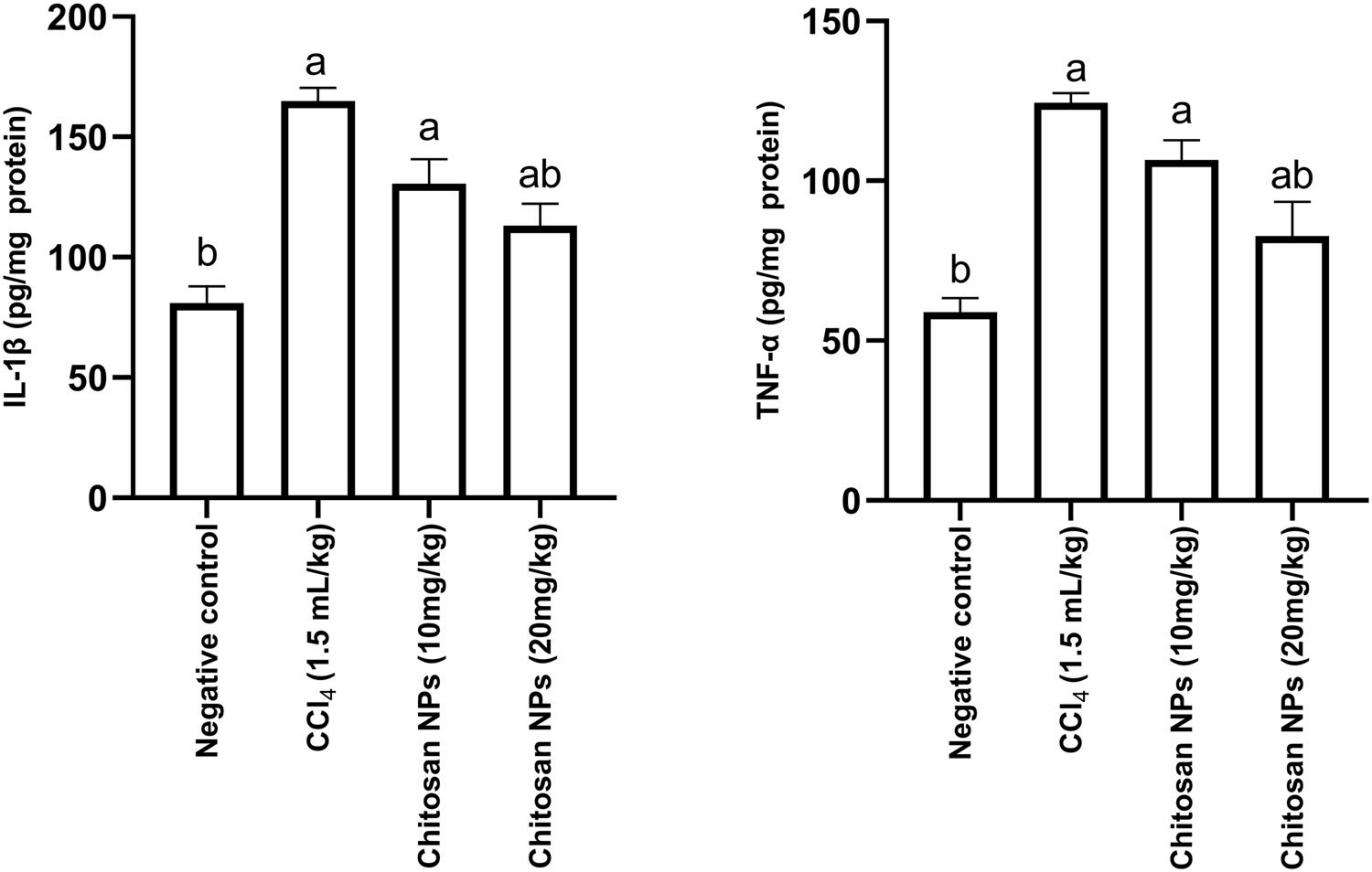
Effect of chitosan NPs (10 and 20 mg/kg) on tissue IL-1β and TNF-α concentrations in CCl_4_-induced nephrotoxicity in rats. Data are expressed as mean ± SD. Statistical analyses were performed by one-way ANOVA, followed by Tukey’s *post hoc* test. ^a^*p* < 0.05: statistically significant from the negative control group and ^b^*p* < 0.05: statistically significant from the CCl_4_ control group. CCl_4_: carbon tetrachloride; IL-1β: interleukin-1β; TNF-α: Tumour necrosis factor-α.

IL-1 and TNF-α are cytokines that are necessary for initiating the innate immune response, mediating the recruitment, activation, and adherence of circulating phagocytic cells (macrophages and neutrophils). For therapeutic strategies, recent studies have been focussed on cytokines modulation (Haddad 2002). IP injection of CCl_4_ resulted in a significant (*p* < 0.0001) increase in renal IL-1β concentration (166.6 ± 9.2 pg/mg protein) when compared with the negative control group (80.9 ± 2.9 pg/mg protein). Only the group that received chitosan NPs (20 mg/kg) had a significant (*p* < 0.0001) decrease in IL-1β concentration (114.8 ± 15.3 pg/mg protein) when compared with the positive control group. A similar response was observed for TNF-α. IP injection of CCl_4_ over 14 days resulted in a significant (*p* < 0.0001) increase in renal TNF-α concentration (124.4 ± 1.2 pg/mg protein) when compared with the negative control group (58.9 ± 1.8 pg/mg protein). Only the group that received chitosan NPs (20 mg/kg) had a significant (*p* < 0.0001) decrease in TNF-α concentration (82.7 pg/mg protein) when compared with the positive control group (Figure 6).

### Determination of apoptotic biomarkers (caspase-3)

**Figure 7.**
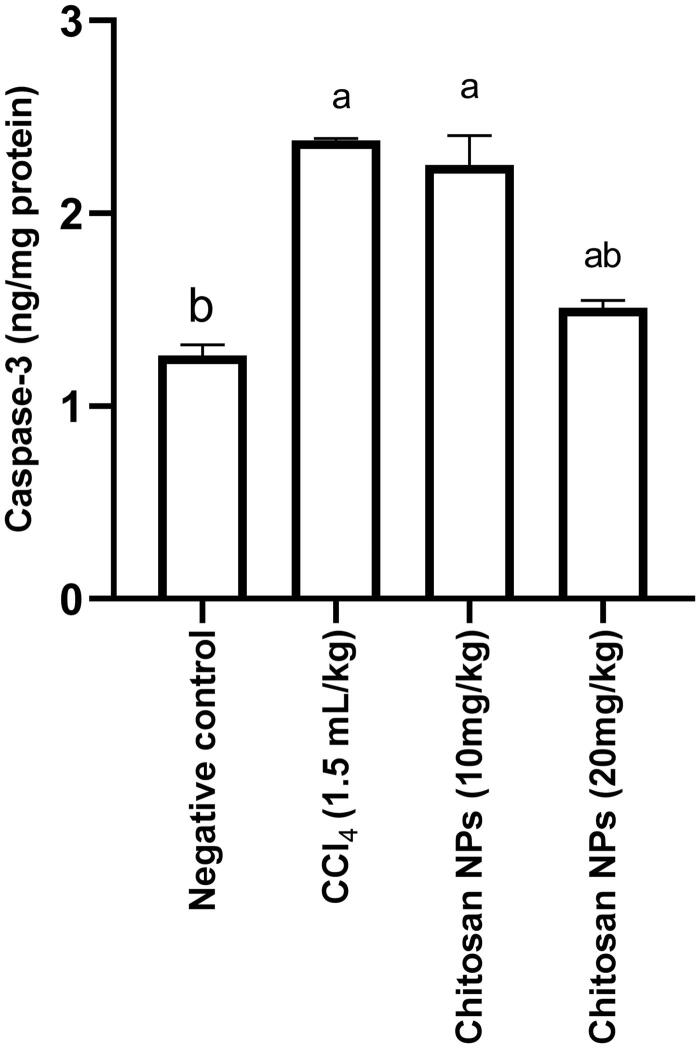
Effect of chitosan NPs (10 and 20 mg/kg) on the apoptotic marker, caspase-3 in CCl_4_-induced rat model of nephrotoxicity. All data are presented as mean ± SD (*n* = 6), ^a^ *p* < 0.05 denotes statistical significance. ^a^ *p* < 0.05 compared with the negative control group and ^b^*p* < 0.05 vs. CCl_4_ group. CCl_4_, carbon tetrachloride.

Caspase-3 is an enzyme that plays a crucial role in programmed cell death (apoptosis) (Grütter 2000). Inhibition of caspase-3 enzyme is considered as evidence of antiapoptotic effect of the tested compound. In the current study, IP injection of CCl_4_ resulted in a significant (*p* < 0.0001) increase in renal caspase-3 concentration (2.4 ± 0.009 ng/mg protein) when compared with the negative control group (1.3 ± 0.055 ng/mg protein). Only the group that received chitosan NPs (20 mg/kg) had a significant (*p* < 0.0001) decrease in caspase-3 concentration (1.5 ± 0.038 nmol/mg protein) when compared with the CCl_4_ control group (Figure 7).

### Histopathological studies

**Figure 8.**
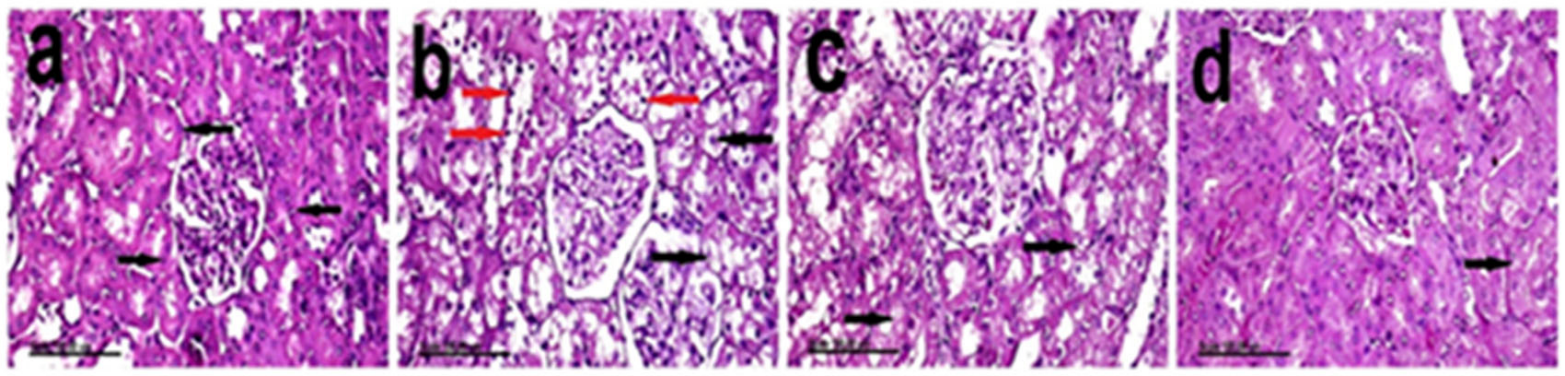
Kidneys of (A) negative control rats showing normal renal tubules, normal round vesicular nuclei (arrows) and renal glomeruli, (B) CCl_4_-treated rats showing swelling of epithelial lining of renal tubules, extensive vacuolation of their cytoplasm (black arrows) and pyknosis of their nuclei that appeared small and intensely basophilic (red arrows), (C) chitosan NPs (10 mg/kg) group showing mild vacuolation and decreased swelling of renal tubules (arrows), and (D) chitosan NPs (20 mg/kg) group showing mild vacuolation of the individual renal tubular epithelium (arrow) (stain: H&E, scale bar = 100 µm).

Kidneys of the normal control group showed normal histological structure, normal renal tubules and rounded vesicular nuclei (Figure 8(A)). On the contrary, kidneys of the CCl_4_-treated group revealed marked swelling of the epithelial lining of renal tubules, extensive vacuolation of their cytoplasm and pyknosis of their nuclei that appeared small and intensely basophilic (Figure 8(B)). Marked attenuation of the histopathological lesions was recorded in the chitosan NPs (10 mg/kg) group, in which mild vacuolation of renal tubules and no evidence of nuclear pyknosis were demonstrated (Figure 8(C)). Pronounced amelioration, with a significant decrease in the pathologic lesion, was demonstrated in the group that received chitosan NPs (20 mg/kg), in which renal tubules appeared normal in most of the examined sections, and only mild vacuolation of the individual renal tubular epithelium was observed (Figure 8(D)).

### Immunohistochemical studies

**Figure 9.**
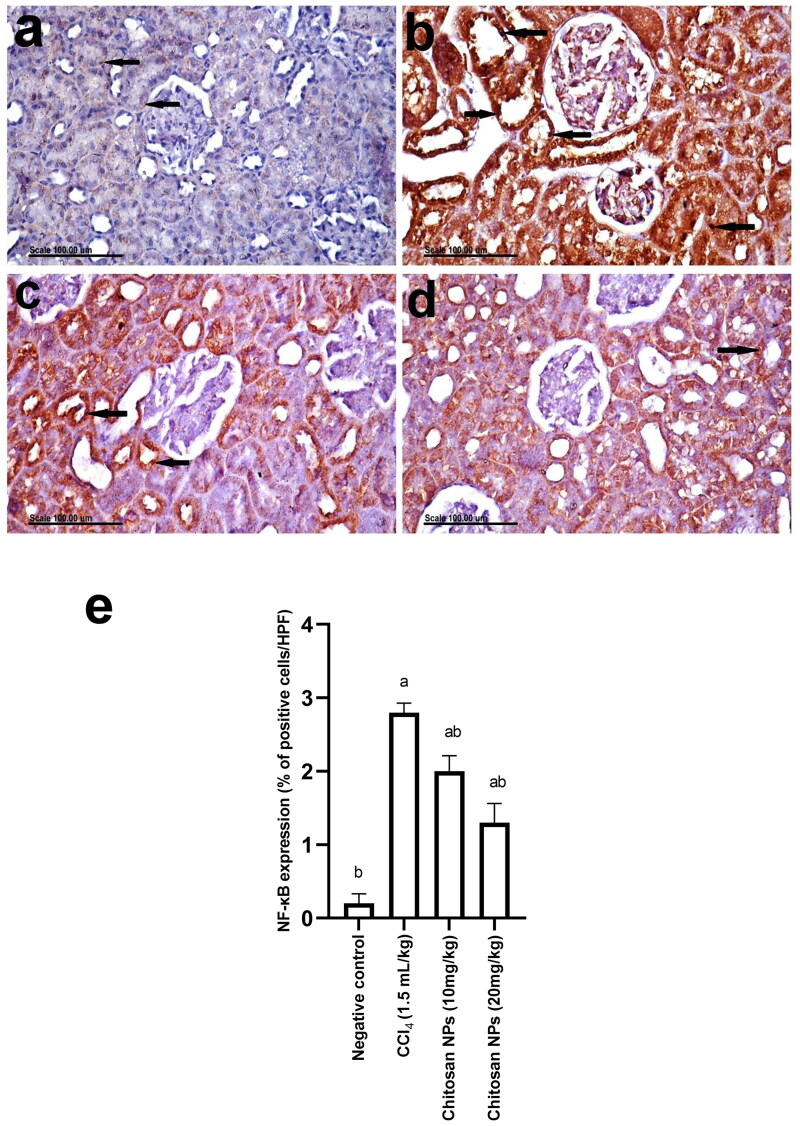
Photomicrographs showing NF-κB immunohistochemical expression in the kidneys of (A) normal control rats showing normal weak cytoplasmic staining of epithelial cells lining renal tubules without nuclear staining (arrow), (B) CCl_4_-treated rats showing a significant increase in NF-κB expression with strong nuclear staining of renal tubules (arrows), (C) chitosan NPs (10 mg/kg) group showing a significant decrease in NF-κB expression and a decreased percentage of positive cells with positive nuclear staining in renal tubules (arrows), (D) chitosan NPs (20 mg/kg) group showing nuclear staining in renal tubules and normal cytoplasmic staining (arrow), and (E) the bar chart of the percentage of the positively stained cells in the different groups. Scale bar =100 µm.

The results of NF-κB immunohistochemical expression recorded in kidneys of normal and treated groups are demonstrated in Figure 9. Kidneys of the normal control group showed normal cytoplasmic staining of epithelial cells lining renal tubules without nuclear staining (Figure 9(A)). On the contrary, a significant increase in NF-κB expression was observed in kidneys of the CCl_4_-treated group, in which nuclear translocation with strong nuclear staining was demonstrated (Figure 9(B)**)**. On the other hand, a significant decrease in NF-κB expression and a decreased percentage of positive cells with positive nuclear staining were observed in the kidneys of the chitosan NPs (10 mg/kg) group (Figure 9(C)). A significant difference was recorded in the chitosan NPs (20 mg/kg) group, in which nuclear staining was demonstrated in individual renal tubular epithelial cells (Figure 9(D)). Figure 9(E) represents the bar chart of the percentage of the positively stained cells in the different groups.

**Figure 10.**
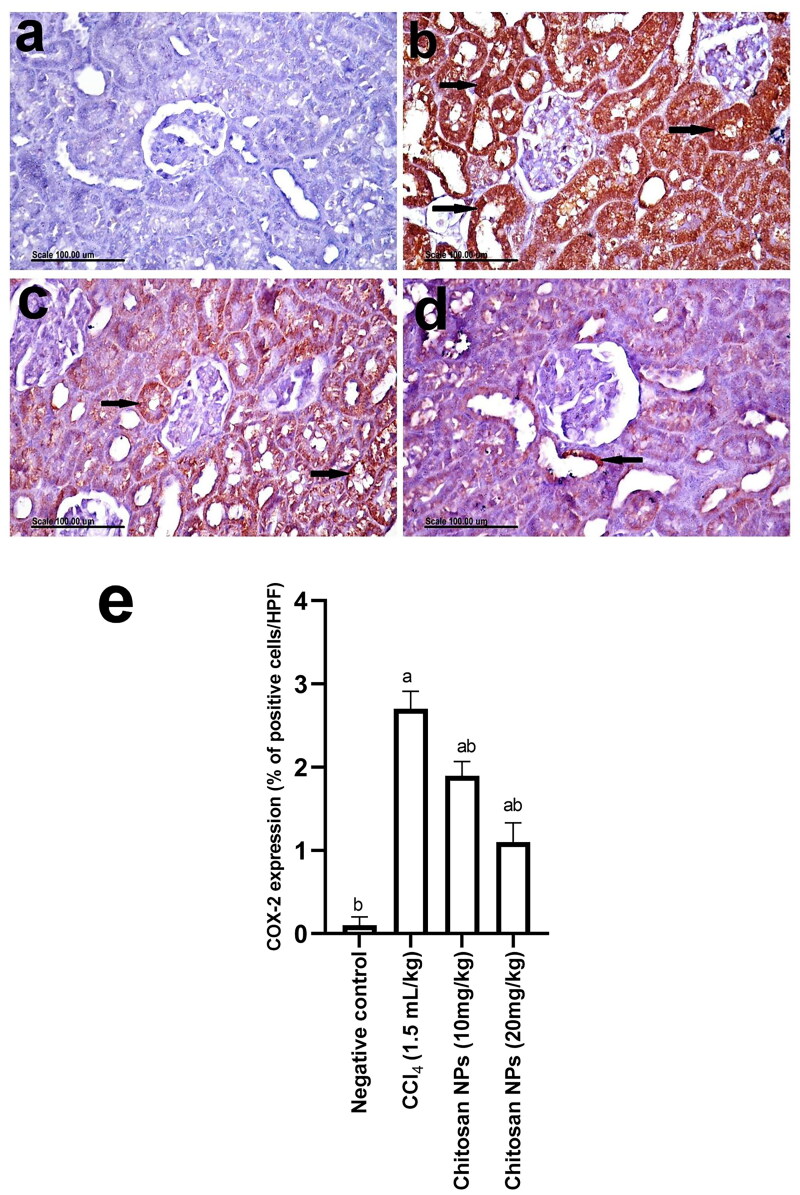
Photomicrographs showing COX-2 immunohistochemical expression in the kidneys of (A) normal control rats showing no COX-2 expression the kidneys, (B) CCl_4_-treated rats showing an increased percentage of positively stained cells with strong cytoplasmic staining of the renal tubular epithelium (arrows), (C) chitosan NPs (10 mg/kg) group showing the decreased percentage of positively stained renal tubular epithelium (arrows), (D) chitosan NPs (20 mg/kg) group showing few COX-2 positively stained cells in the renal tubular epithelium (arrow), and (E) the bar chart of the percentage of the positively stained cells in the different groups. Scale bar = 100 µm.

The results of COX-2 immunohistochemical expression in the kidneys of normal and treated groups are demonstrated in Figure 10. No COX-2 expression was observed in the kidneys of the normal control group (Figure 10(A)). In contrast, a significant increase in COX-2 expression was recorded in kidneys of the CCl_4_-treated group, which showed an increased percentage of positively stained cells with strong cytoplasmic staining of the renal tubular epithelium (Figure 10(B)). Additionally, COX-2 expression was also demonstrated in the infiltrating inflammatory cells. Treatment with chitosan NPs showed a significant decrease in COX-2 expression in the kidneys in a dose-dependent manner, where a significant difference between the two doses was observed (Figure 10(C, D)). Figure 10(E) represents the bar chart of the percentage of the positively stained cells in the different groups.

## Discussion

The current study describes the preparation of chitosan NPs via ionotropic gelation and formation of polyelectrolyte complexes between chitosan and sodium alginate. The prepared NPs displayed nanosized assemblies of hydrodynamic diameter 56 ± 18 nm and homogenous size distribution (PDI = 0.41 ± 0.05), as determined by dynamic light scattering. Positive zeta potential of *ca.* 32.5 ± 1.4 mV of the formed particles implies good stability and low tendency of aggregation due to electrostatic repulsion between the positively charged particles. The TEM image of the chitosan NPs demonstrates the formation of globular nanosized particles of moderate size distribution, in agreement with the dynamic light scattering data.

The interaction between chitosan and sodium alginate was characterized by FTIR spectroscopy. The chitosan spectrum revealed peaks that correspond to their functional groups. The interference between the NH and OH groups was confirmed via the NH stretching peaks. The spectrum of sodium alginate demonstrated peaks corresponding to its functional groups, where the OH group bond was dominating with a wide peak. The shift of bands in the processed NPs and the change in intensities of peaks provides evidence for the formation of NPs. Thermogram of chitosan indicates an initial endothermic peak followed by a broad exothermic peak. The appearance of the endothermic peak is due to the water deprivation of water linked to hydrophilic groups of polymers indicating that chitosan was partially hydrated, and water was not fully removed during dryness while the exothermic peak was correlated to degradation of chitosan through dehydration and depolymerization reactions due to the partial decarboxylation of COOH groups and oxidation reactions of the polyelectrolytes. The DSC of sodium alginate showed an initial endothermic peak and a higher exothermic peak. This thermogram agrees with the melting and breakdown of pure sodium alginate, respectively, and similar profile was reported previously (Singh 2013). The disappearance of characteristic peaks and the shifting of endothermic peaks with higher intensity of chitosan and sodium alginate in the thermogram of chitosan NPs revealed the interaction between the two polymer-forming polyelectrolyte complexes. This proves the good interaction between chitosan and alginate and the formation of polyelectrolyte complex with good firmness.

CCl_4_ is one of the numerous environmental toxicants that are implicated in various detrimental cellular damage in different body organs (Noguchi et al. 1982). CCl_4_ is considered a reference drug for induction of organ toxicities, such as nephrotoxicity, cardiotoxicity, and hepatotoxicity, in experimental animal models (Islam et al. 2002). The CCl_4_ toxicity was ascribed to its metabolic activation via the P450 system to generate trichloromethyl and chloride radicals which are highly reactive free radicals that possess high affinity to bind to electrons in organ tissues, thus leading to protein peroxidation and DNA impairment (Alkreathy et al. 2014). Induced renal dysfunction via CCl_4_ treatment was previously thought to be due to the liver functional condition or it may occur independently to hepatic state (Rincón et al. 1999). It has been also reported that the distribution of CCl_4_ in renal tissue is much higher than in liver tissue (Sanzgiri and Bruckner 1997). In a previous study, the nephrotoxicity in rats was attributed to oxidative stress induced by CCl_4_ (Abraham et al. 1999). It was also reported that kidney damage due to CCl_4_ exposure was due to the generation of reactive oxygen species (Ganie et al. 2011). In the current study, the possible ameliorative effect of chitosan NPs at two doses (10 and 20 mg/kg) against CCl_4_-induced nephrotoxicity in rats, was investigated.

Our study revealed that injection of CCl_4_ significantly elevated creatinine serum level, which is a nitrogenous end product of metabolism that is removed from the blood through the kidney. This function was impaired after CCl_4_ injection, and excretion of the creatinine from blood was reduced, as evidenced by the markedly increased level in the blood. The results are in agreement with previously reported data in the literature (Elsawy et al. 2019). This increase might be attributed to the injury that occurred to the nephron structural integrity (Makni et al. 2012). The group injected with CCl_4_ showed a marked swelling of the epithelial lining renal tubules that was associated with extensive vacuolation of their cytoplasm and pyknosis of their nuclei that appeared small and intensely basophilic. Concurrent administration of chitosan NPs resulted in a significant decrease in creatinine serum level which could be attributed to the pronounced amelioration of pathological lesions in which the renal tubules appeared normal. This effect may be attributed to the free radical scavenging of chitosan NPs reported previously by our group (El-Shafei et al. 2021).

It was reported that CCl_4_ generates free radicals in the tissues of the liver, kidney, brain, and lungs (Hamed et al. 2012). These free radicals result in protein denaturation, lipid peroxidation, and cell death (Hismiogullari et al. 2015). Lipid peroxidation and GSH are vital biomarkers for the assessment of oxidative stress (Firuzi et al. 2011). Glutathione is an endogenous antioxidant that acts by inhibiting the injury of cellular components via scavenging of lipid peroxides and free radicals (Kaur et al. 2003). Our findings revealed that administration of CCl_4_ caused marked oxidative stress which was evidenced by pronounced elevation of MDA and depletion of reduced GSH, as reported earlier (Suzuki et al. 2015). The significant depletion of GSH was attributed to the elevation of hydrogen peroxide levels in the renal cortex and medulla which is implicated in oxidative stress (Gomes et al. 2009). Oral administration of chitosan NPs at both dose levels showed a significant decrease in MDA as well as a remarkable increase in GSH which indicates a prominent antioxidant effect of chitosan NPs against CCl_4_-induced oxidative stress. In a previous study, chitosan revealed a protective effect against lead-induced oxidative stress in rats (Özdek et al. 2019).

A cross-talk was observed in the current study between oxidative stress and inflammatory cytokines, as CCl_4_ administration showed a remarkable increase in inflammatory cytokines (IL-1β and TNF-α). This relationship could be attributed to the generation of trichloromethyl and trichloromethyl peroxyl radicals triggered by CCl_4_. The increase in oxidative stress might enhance inflammatory cytokines burst via different mechanisms. It was reported that oxygen derivatives activate NF-κB and activator protein-1 (AP-1), which led to the transcription of genes that encode inflammatory cytokines. NF- κB plays a vital role in mesangial cell activation causing renal injury (Elmarakby and Sullivan 2012). Our findings showed that chitosan NPs at low and high doses significantly reduced the elevated inflammatory cytokines, probably due to the suppression of oxidative stress.

The effect of CCl_4_ on NF-κB was confirmed by an immunohistochemical study that demonstrated a significant increase in NF-κB expression in kidneys, in which nuclear translocation with strong nuclear staining was observed. Ranneh et al. (2017) reported that the activation of NF- κB upregulates the gene expression of COX-2. Similarly, in our study, we observed an increase in COX-2 expression in kidneys of the CCl_4_-treated group. The administration of chitosan NPs resulted in a remarkable amelioration in the expression of both NF-κB and COX-2 in renal tissues.

Apoptosis is a process of programmed cellular death (Coles et al. 1993). In normal conditions, apoptosis rarely occurs in the kidney of adults as cellular turnover is low (Conaldi et al. 1998). Kidney injury exhibits a pronounced increase in apoptotic cells to remove damaged cells that failed to repair (Zager and Burkhart 1998). The caspase-3 enzyme, also known as executioner caspase, is an endoprotease that orchestrates the apoptosis signalling cascade. It is produced in an inactive proform that is activated via caspase-9, caspase 8, and granzyme B to generate the active caspase-3 (Elmore 2007). In the current research, we reported a significant increase in caspase-3 levels in renal tissues in the group treated with CCl_4_ indicating cellular damage and apoptosis. Our findings are in agreement with that of Hassan et al. (2015) and Hwang et al. (2013). Oral administration of chitosan NPs significantly reduced caspase-3 levels thus revealing an antiapoptotic effect that might result from inhibition of caspase-3 activation.

## Conclusions

Chitosan NPs were prepared via the ionotropic gelation method, and displayed globular nanosized particles of homogenous distribution. Chitosan NPs (20 mg/kg) improved renal function and suppressed the oxidative stress, inflammatory and apoptotic effects in CCl_4_-induced nephrotoxicity rat model. Pathological and immunohistochemical studies showed a pronounced recovering effects of kidney tissue upon concurrent administration of chitosan NPs over 2 weeks. Overall, the potential underlying mechanisms of the chitosan NPs to ameliorate CCl_4_-induced nephrotoxicity were by suppressing oxidative stress, inhibition of inflammatory cytokines as well as deactivation of the caspase-3 enzyme that resulted in antioxidant, anti-inflammatory, and antiapoptotic effects. Hence, chitosan NPs could afford a potential nanotherapeutic system for the management of nephrotoxicity.
